# Can the use of a visual obturator-assisted resectoscope reduce the incidence of urethral strictures after TURP?: a multicenter propensity-matched study

**DOI:** 10.1007/s00345-026-06558-z

**Published:** 2026-06-23

**Authors:** Muhammed Selcuk Ozer, Volkan Sen, Alper Ege Sarikaya, Mehmed Emin Kayacan, Murat Gulsen, Murat Isitan, Nebil Akdogan, Laith Abedalqader Abdul Hadi Albanna, Cemil Aydin, Alpkon Torun, Mehmet Emin Arayıcı, Ozan Bozkurt

**Affiliations:** 1 Faculty of Medicine, Department of Urology, Dokuz Eylul University, Izmir, Turkey; 2 Faculty of Medicine, Department of Urology, Ondokuz Mayis University, Samsun, Turkey; 3 Faculty of Medicine, Department of Urology, Cukurova University, Adana, Turkey; 4 Faculty of Medicine, Department of Urology, Hitit University, Corum, Turkey; 5 Faculty of Medicine, Department of Biostatistics, Dokuz Eylul University, Izmir, Turkey

**Keywords:** Benign prostatic hyperplasia, Transurethral resection of prostate, Urethral stricture, Surgical instruments, Postoperative complications

## Abstract

**Purpose:**

Urethral stricture (US) is a significant long-term complication of transurethral resection of the prostate (TURP), often linked to mechanical trauma during instrument insertion. This study evaluated whether advancing the resectoscope sheath under direct vision using a visual obturator reduces postoperative US incidence compared to blind insertion.

**Materials and methods:**

A retrospective multicenter study was conducted across four centers involving patients who underwent bipolar TURP between 2020 and 2025. After applying propensity score matching to minimize baseline bias, 962 patients were analyzed (481 per group: visual vs. blind obturator). Primary outcomes included US incidence, perioperative variables, and functional results (Qmax). Subgroup analysis was performed based on prostate volume.

**Results:**

The overall US incidence was 12.4%. Patients in the visual obturator group had a significantly lower US rate compared to the blind group (7.7% vs. 17.0%, *p* < 0.001). Visual guidance also correlated with a significant reduction in overall postoperative complications and acute urinary retention. Subgroup analysis revealed that the protective effect of the visual obturator was most pronounced in prostates > 60 cc. While traditional predictors like operative time and prostate volume showed modest discriminatory ability for US risk in the blind group, their impact was mitigated in the visual group. Both groups showed improved Qmax, with a higher baseline and more stable trend in the visual cohort.

**Conclusion:**

Direct vision advancement of the resectoscope sheath using a visual obturator significantly reduces the risk of urethral stricture and postoperative morbidity. This technique offers a simple, effective strategy to minimize urethral trauma, particularly in larger prostates, and should be considered for routine use.

## Introduction

Benign prostatic hyperplasia (BPH) is the most common urological condition affecting middle-aged and elderly men, frequently leading to lower urinary tract symptoms (LUTS) [1–5]. Transurethral resection of the prostate (TURP) has long been used in the surgical management of LUTS caused by bladder outlet obstruction (BOO) and continues to be regarded as the “gold standard” treatment modality [1–8]. However, postoperative complications such as urethral stricture (US) and bladder neck contracture (BNC) represent significant long-term morbidities that may impair surgical efficacy and substantially reduce patients’ quality of life [1, 3, 5, 6].

The incidence of US following TURP has been reported to range between 4.5% and 13% in the literature [1, 3, 4]. Although the exact etiology of US remains unclear, the predominant mechanisms are believed to involve intraoperative urethral mucosal injury, subsequent scar formation, and persistent inflammation [1, 4]. Several perioperative risk factors contributing to stricture formation have been identified, including low resection speed, intraoperative mucosal rupture, persistent postoperative infection, prolonged catheterization, and the diameter/caliber of the resectoscope [1, 3, 8].

Mechanical trauma is widely accepted as a major contributor to stricture development; such trauma may result from an inadequately sized resectoscope or from repeated “in-and-out” movements during the procedure [3, 4, 9]. Because intraoperative urethral mucosal injury has been demonstrated to be an independent risk factor for US, the initial insertion of surgical instruments into the urethra is considered a critical step in minimizing potential trauma [1, 2, 9].

To prevent US after TURP, several precautionary measures have been suggested, including thorough lubrication of the cystoscope sheath, careful selection of sheath size, and advancement of the instrument under direct visualization using a visual obturator [1, 3, 8]. These recommendations imply that advancing the resectoscope with a “blind obturator” may increase the risk of mechanical trauma to the urethra. In this context, our study evaluates whether the two commonly used techniques for introducing the resectoscope sheath (advancement with a blind obturator versus advancement under direct vision using a visual obturator) differ in terms of postoperative US incidence.

Considering the potential differences in mechanical stress and mucosal trauma associated with these two approaches, determining their impact on US development may offer clinically relevant insights to enhance procedural safety and reduce complications in TURP. In particular, we investigate whether visual guidance during instrument insertion provides superior protection against mucosal injury, a well-established risk factor for stricture formation.

## Materials and methods

Between November 2020 and June 2025, patients who underwent bipolar TURP for BPH in four tertiary referral centers were retrospectively reviewed. To avoid the influence of sheath caliber as a confounding factor in US development, the study was restricted to patients who underwent surgery using a 26 Fr resectoscope sheath. Patients with a postoperative pathology result of prostate adenocarcinoma, those with a history of pelvic radiotherapy, and those diagnosed with urethral stricture prior to TURP were excluded from the study. The study was conducted in accordance with the Declaration of Helsinki and was approved by the Ethics Committees of the participating institutions (Dokuz Eylul University approval number: 2023/05–23).

Demographic characteristics (age, BMI, comorbidities, smoking habits), prostate volume, PSA levels, digital rectal examination findings, uroflowmetry parameters, surgical details (operative time, intraoperative complications, and amount of resected tissue), as well as postoperative follow-up data (uroflowmetry results, postoperative complications, and presence of urethral stricture) were retrospectively collected from the electronic medical records of all four centers. Patients were then divided into two groups based on whether a visual obturator was used during surgery, and the groups were compared accordingly.

### Sample size calculation and power analysis

A priori sample size estimation was performed using G*Power 3.1 for an independent samples t test (two-tailed, medium effect size, alpha = 0.05, power = 0.80, equal group allocation). The required sample size was 326 patients per group (total *n* = 652), ensuring adequate statistical power to detect the prespecified effect size.

### Statistical analysis

To minimize baseline imbalance between patients treated with and without a visual obturator, propensity score matching (PSM) was applied. Propensity scores were derived from a logistic regression model including prostate volume, bladder stones, and operation time. One-to-one nearest-neighbor matching without replacement was performed using a caliper of 0.2 SD of the logit. Covariate balance was assessed by standardized mean differences (< 0.10 considered acceptable).

Continuous variables were expressed as mean ± SD and categorical variables as counts and percentages. Normality was tested with Shapiro–Wilk. Between-group comparisons were performed using independent samples t test for continuous variables and chi-square or Fisher’s exact test for categorical variables. Subgroup analyses stratified by prostate volume ( < = 30, 31–60, 61–90, > 90 cc) were conducted to explore effect modification. Longitudinal changes in maximum urinary flow rate (Qmax) were evaluated with paired t tests, and effect sizes were quantified using Cohen’s d.

Receiver operating characteristic (ROC) analyses were performed to assess the discriminative ability of clinical variables for urethral stricture, with AUCs and 95% CIs calculated nonparametrically. Optimal cutoffs were determined using the Youden index. All statistical analyses were undertaken using IBM SPSS Statistics (version 30.0; IBM Corp., Armonk, NY, USA) and STATA (version 16; StataCorp LLC, College Station, TX, USA). All tests were two-sided, and a p value < 0.05 was considered statistically significant.

## Results

The study cohort consisted of 962 patients, divided equally into two groups after propensity matching: those who underwent procedures with a visual obturator (*n* = 481) and those who did not (*n* = 481) (Table 1). The mean age of the overall population was 69.7 ± 9.2 years, with no statistically significant difference observed between the groups (*p* = 0.859), indicating a well-matched age distribution. Similarly, operation time (*p* = 0.720) and prostate volume (*p* = 0.074) remained comparable across both cohorts.

Body mass index (BMI) was slightly lower in the visual obturator group (26.56 ± 3.60 vs. 27.01 ± 3.47 kg/m²; *p* = 0.049), whereas smoking exposure was substantially higher among patients treated with a visual obturator (22.84 ± 24.21 vs. 11.19 ± 15.92 pack–years; *p* < 0.001). Prostate volume distribution across predefined categories did not differ significantly between groups (*p* = 0.179). Similarly, the prevalence of bladder stones and major comorbidities was comparable between groups.

**Table 1:** Baseline demographic and clinical characteristics according to visual obturator use (*n* = 962)

Variable	Overall (*n* = 962)	Without visual obturator (*n* = 481)	With visual obturator (*n* = 481)	*p* value
Age, years	69.72 ± 9.28	69.67 ± 8.69	69.77 ± 9.84	0.859*
Operation time, min	67.70 ± 27.72	67.38 ± 26.43	68.02 ± 28.95	0.720*
Body mass index, kg/m²	26.79 ± 3.54	27.01 ± 3.47	26.56 ± 3.60	0.049*
Prostate volume, cc	67.66 ± 33.71	65.72 ± 29.24	69.60 ± 37.66	0.074*
Smoking exposure, pack–years	17.02 ± 20.49	11.19 ± 15.92	22.84 ± 24.21	< 0.001*
Stricture	119 (12.4%)	82 (17%)	37 (7.7%)	< 0.001*
Prostate volume category				0.179**
0–30 cc	87 (9.0%)	37 (7.7%)	50 (10.4%)	
31–60 cc	402 (41.8%)	210 (43.7%)	192 (39.9%)	
61–90 cc	300 (31.2%)	156 (32.4%)	144 (29.9%)	
	173 (18.0%)	78 (16.2%)	95 (19.8%)	
Current smoking	498 (51.8%)	183 (38.0%)	315 (65.5%)	< 0.001**
Urethral stricture	119 (12.4%)	82 (17.0%)	37 (7.7%)	< 0.001**
Bladder stone	84 (8.7%)	42 (8.7%)	42 (8.7%)	0.999**
Postoperative complication	131 (13.6%)	90 (18.7%)	41 (8.5%)	< 0.001**
Acute urinary retention	177 (18.4%)	167 (34.7%)	10 (2.1%)	< 0.001**
Chronic kidney disease	41 (4.3%)	21 (4.4%)	20 (4.2%)	0.873**
Hypertension	496 (51.6%)	245 (50.9%)	251 (52.2%)	0.699**
Diabetes mellitus	243 (25.3%)	130 (27.0%)	113 (23.5%)	0.207**
Coronary artery disease	264 (27.4%)	140 (29.1%)	124 (25.8%)	0.248**
Chronic obstructive pulmonary disease	68 (7.1%)	30 (6.2%)	38 (7.9%)	0.314**
Congestive heart failure	11 (1.1%)	7 (1.5%)	4 (0.8%)	0.363**
Hypothyroidism	47 (4.9%)	14 (2.9%)	33 (6.9%)	0.004**
Hyperthyroidism	4 (0.4%)	1 (0.2%)	3 (0.6%)	0.305***
Intraoperative complication	24 (2.5%)	23 (4.8%)	1 (0.2%)	< 0.001**

Continuous variables are presented as mean ± standard deviation and categorical variables as number (percentage)

*Independent samples t test. **Pearson χ² test. ***Fisher’s exact test. A two-sided p value < 0.05 was considered statistically significant

The overall prevalence of urethral stricture was 12.4%, with a significantly lower rate observed in the visual obturator group compared with the blind obturator group (7.7% vs. 17.0%; *p* < 0.001) (Table 1). Acute urinary retention occurred less frequently in the visual obturator group (2.1% vs. 34.7%; *p* < 0.001), and postoperative complications were also significantly reduced (8.5% vs. 18.7%; *p* < 0.001). Intraoperative complications were rare overall but were observed more frequently in patients undergoing procedures without a visual obturator (4.8% vs. 0.2%; *p* < 0.001).

As seen in Table 2, subgroup analyses stratified by prostate volume demonstrated an association between visual obturator use and a lower incidence of urethral stricture across increasing prostate size categories. In patients with small prostates (0–30 cc), urethral stricture rates were numerically lower in the visual obturator group compared with the blind obturator group (6.0% vs. 13.5%; *p* = 0.277). A similar trend was observed in the 31–60 cc subgroup, where stricture rates were 7.8% in the visual obturator group versus 13.8% in the blind obturator group (*p* = 0.054).

In contrast, among patients with larger prostate volumes, the protective association of visual obturator use became statistically significant. In the 61–90 cc subgroup, urethral stricture occurred significantly less frequently in patients treated with a visual obturator compared with those without (8.3% vs. 21.2%, *p* = 0.002). This effect persisted in the > 90 cc subgroup, where visual obturator use was associated with a substantially lower stricture rate (7.4% vs. 19.2%, *p* = 0.020).

**Table 2:** Subgroup analysis of the association between visual obturator use and urethral stricture across prostate volume categories

Prostate volume category	Visual obturator	Stricture no *n* (%)	Stricture yes *n* (%)	χ² (df)	*p* value
0–30 cc (*n* = 87)	No	32 (86.5)	5 (13.5)	1.44 (1)	0.277**
	Yes	47 (94.0)	3 (6.0)		
31–60 cc (*n* = 402)	No	181 (86.2)	29 (13.8)	3.70 (1)	0.054*
	Yes	177 (92.2)	15 (7.8)		
61–90 cc (*n* = 300)	No	123 (78.8)	33 (21.2)	9.65 (1)	0.002*
	Yes	132 (91.7)	12 (8.3)		
> 90 cc (*n* = 173)	No	63 (80.8)	15 (19.2)	5.43 (1)	0.020*
	Yes	88 (92.6)	7 (7.4)		

*Fisher’s exact test. **χ² statistics correspond to Pearson chi-square tests (2 × 2 tables). Values are presented as number (percentage within visual obturator category). All tests are two-sided; *p* < 0.05 was considered statistically significant. No cells violated minimum expected count assumptions

Among patients treated without a visual obturator, baseline continuous variables did not differ significantly according to urethral stricture status (Table 3). Patients who developed urethral stricture had a mean age of 70.45 ± 8.01 years compared with 69.51 ± 8.83 years in those without stricture (*p* = 0.371). Mean operation time was longer in patients with stricture (72.44 ± 25.27 min vs. 66.35 ± 26.57 min), although this difference did not reach statistical significance (*p* = 0.051; Cohen’s d = − 0.23).

**Table 3:** Association, group comparisons, and longitudinal outcomes related to urethral stricture according to visual obturator use

Baseline continuous variables by stricture status															
Visual obturator	Variable		Stricture No (Mean ± SD)			Stricture yes (Mean ± SD)			Mean difference		*p* value			Effect size (Cohen’s d)	
No	Age (years)		69.51 ± 8.83			70.45 ± 8.01			−0.95		0.371			−0.11	
	Operation time (min)		66.35 ± 26.57			72.44 ± 25.27			−6.09		0.051			−0.23	
	BMI (kg/m²)		27.04 ± 3.60			26.86 ± 2.78			0.19		0.659			0.05	
	Smoking (pack–years)		11.12 ± 16.10			11.52 ± 15.11			−0.41		0.832			−0.03	
	Prostate volume (cc)		64.73 ± 29.12			70.54 ± 29.55			−5.81		0.101			−0.20	
Yes	Age (years)		69.72 ± 9.87			70.41 ± 9.61			−0.69		0.685			−0.07	
	Operation time (min)		67.89 ± 29.10			69.59 ± 27.45			−1.70		0.732			−0.06	
	BMI (kg/m²)		26.51 ± 3.57			27.16 ± 3.85			−0.65		0.290			−0.18	
	Smoking (pack–years)		23.17 ± 24.17			18.92 ± 24.64			4.25		0.305			0.18	
	Prostate volume (cc)		69.75 ± 38.14			67.78 ± 31.63			1.97		0.760			0.05	

Categorical outcomes associated with stricture															
Visual obturator		Variable			Stricture No (*n* = 843)			Stricture Yes (*n* = 119)							*p-value*
No					(*n* = 399)			(*n* = 82)							
		Bladder Stone, n (%)			34 (8.5)			8 (9.8)							0.718
		AUR, n (%)			141 (35.3)			26 (31.7)							0.529
		Postop Complications, n (%)			8 (2.0)			82 (100)							< 0.001
Yes					(*n* = 444)			(*n* = 37)							
		Bladder Stone, n (%)			39 (8.8)			3 (8.1)							1.000
		AUR, n (%)			10 (2.3)			0 (0.0)							1.000
		Postop Complications, n (%)			4 (0.9)			37 (100)							< 0.001

Longitudinal changes in Qmax															
Comparison		Visual obturator		Time point			Mean ± SD (mL/s)			Mean difference		95% CI	*p* value		Effect size (Cohen’s d)
Preop → 1st month		No		Preoperative			7.64 ± 3.46			−6.63		−7.15 to − 6.11	< 0.001		−1.14
				1st month			14.26 ± 4.34								
Preop → 6th month		No		Preoperative			7.64 ± 3.46			−5.06		−5.63 to − 4.50	< 0.001		−0.80
				6th month			12.70 ± 5.49								
1st → 6th month		No		1st month			14.26 ± 4.34			1.57		1.04 to 2.10	< 0.001		0.26
				6th month			12.70 ± 5.49								
Preop → 1st month		Yes		Preoperative			8.10 ± 3.78			−3.53		−4.17 to − 2.88	< 0.001		−0.49
				1st month			11.63 ± 6.81								
Preop → 6th month		Yes		Preoperative			8.10 ± 3.78			−4.54		−5.17 to − 3.92	< 0.001		−0.65
				6th month			12.64 ± 6.55								
1st → 6th month		Yes		1st month			11.63 ± 6.81			−1.01		−1.41 to − 0.62	< 0.001		−0.23
				6th month			12.64 ± 6.55								

Continuous variables are presented as mean ± standard deviation. Between-group comparisons were performed using independent samples t-tests. Categorical variables were compared using Pearson χ² or Fisher’s exact test, as appropriate. Longitudinal comparisons were assessed using paired samples t-tests with effect sizes. All tests were two-sided; *p* < 0.05 was considered statistically significant

Body mass index, smoking exposure, and prostate volume were also comparable between patients with and without stricture within the blind group. Similar findings were observed in the visual obturator group, where mean age, operation time, body mass index, smoking exposure, and prostate volume did not differ significantly by stricture status (all *p* > 0.05).

Longitudinal analysis of maximum urinary flow rate (Qmax) revealed significant postoperative improvements in both groups. In patients without a visual obturator, mean Qmax increased from 7.64 ± 3.46 mL/s preoperatively to 14.26 ± 4.34 mL/s at the first postoperative month (mean difference − 6.63 mL/s; 95% CI − 7.15 to − 6.11; *p* < 0.001; Cohen’s d = − 1.14) and remained higher at six months (12.70 ± 5.49 mL/s; mean difference − 5.06 mL/s; *p* < 0.001). A modest but statistically significant decline was observed between the first and sixth postoperative months (mean difference 1.57 mL/s; *p* < 0.001). In the visual obturator group, Qmax similarly improved from 8.10 ± 3.78 mL/s preoperatively to 11.63 ± 6.81 mL/s at one month (mean difference − 3.53 mL/s; 95% CI − 4.17 to − 2.88; *p* < 0.001; Cohen’s d = − 0.49) and to 12.64 ± 6.55 mL/s at six months (mean difference − 4.54 mL/s; *p* < 0.001). A small but significant reduction in Qmax was noted between the first and sixth months (mean difference − 1.01 mL/s; *p* < 0.001) (Table 3).

Receiver operating characteristic analyses were performed to evaluate the discriminative ability of selected clinical variables for predicting urethral stricture, stratified by visual obturator use (Table 4). Overall, the discriminatory performance of individual predictors was limited, with most AUC values ranging close to 0.50. In patients treated without a visual obturator, prostate volume demonstrated a modest discriminatory ability for urethral stricture, with an AUC of 0.562 (95% CI 0.494–0.631; *p* = 0.073). In contrast, prostate volume showed no discriminatory value in the visual obturator group (AUC 0.499; 95% CI 0.407–0.590; *p* = 0.975), with a significantly lower Gini index (− 0.003). The between-group difference in AUC was not statistically significant (ΔAUC = 0.064; *p* = 0.273). Operation time exhibited the highest discriminative performance among the evaluated variables in the blind obturator group, with an AUC of 0.574 (95% CI 0.505–0.643; *p* = 0.036). In the visual obturator group, however, operation time was not predictive of urethral stricture (AUC 0.527; 95% CI 0.437–0.616; *p* = 0.563). The difference in AUC between groups was small and not statistically significant (ΔAUC = 0.047; *p* = 0.412). Age demonstrated poor discriminatory performance in both groups. In patients without a visual obturator, the AUC for age was 0.530 (95% CI 0.463–0.597; *p* = 0.381), while in those treated with a visual obturator, the AUC was 0.524 (95% CI 0.419–0.630; *p* = 0.649). The corresponding AUC difference was negligible (ΔAUC = 0.006; *p* = 0.930). Similarly, smoking exposure measured in pack–years did not meaningfully discriminate urethral stricture in either group. In the blind obturator group, the AUC was 0.493 (95% CI 0.425–0.561; *p* = 0.842), whereas in the visual obturator group, the AUC was 0.563 (95% CI 0.470–0.656; *p* = 0.183). The between-group AUC difference did not reach statistical significance (ΔAUC = − 0.070; *p* = 0.233).

**Table 4:** ROC analysis of clinical variables for predicting urethral stricture stratified by visual obturator use

Predictor variable	Visual obturator	AUC	95% CI	Std. error	*p* value†	Optimal cutoff‡	Gini index	Max K–S	ΔAUC (Yes–No)	pΔ
Prostate volume (cc)	No	0.562	0.494–0.631	0.035	0.073	69.5	0.125	0.153	0.064	0.273
	Yes	0.499	0.407–0.590	0.047	0.975	47.5	−0.003	0.081		
Operation time (min)	No	0.574	0.505–0.643	0.035	0.036	69.0	0.148	0.186	0.047	0.412
	Yes	0.527	0.437–0.616	0.046	0.563	67.5	0.053	0.090		
Age (years)	No	0.530	0.463–0.597	0.034	0.381	65.5	0.060	0.106	0.006	0.930
	Yes	0.524	0.419–0.630	0.054	0.649	71.5	0.049	0.122		
Smoking exposure (pack–years)	No	0.493	0.425–0.561	0.035	0.842	32.5	−0.014	0.106	−0.070	0.233
	Yes	0.563	0.470–0.656	0.048	0.183	26.5	0.127	0.155		

ROC analyses were conducted using nonparametric assumptions with 95% confidence intervals. Larger values indicate stronger evidence for urethral stricture, except for smoking pack–years where smaller values indicate higher risk

*AUC* area under the receiver operating characteristic curve

†p value tests the null hypothesis that AUC = 0.50. ‡Optimal cutoff determined by maximum Youden index / Kolmogorov–Smirnov statistic

## Discussion

Benign prostatic hyperplasia (BPH) is one of the most common causes of lower urinary tract symptoms in elderly men [1, 2]. In patients who are refractory to medical therapy, TURP remains the gold standard surgical treatment [1, 3–5]. Nevertheless, urethral stricture (US) following TURP represents a challenging long-term complication leading to repeated interventions and increased patient morbidity [4–7].

Mechanical trauma is widely accepted as a key factor in the development of urethral strictures [3, 5, 6]. Inappropriate selection of the resectoscope sheath or repeated “in–out” movements during surgery may result in urethral mucosal injury, which serves as an independent risk factor for US development [5, 6, 8, 9]. Therefore, the initial insertion of surgical instruments into the urethra represents a critical step of the procedure [1, 6]. Adequate lubrication, appropriate sheath size selection, and advancement of instruments under direct vision have been recommended to minimize urethral injury, whereas blind obturator insertion is considered to increase the risk of mechanical trauma [1, 5, 6].

When the entire cohort was evaluated, the overall incidence of post-TURP urethral stricture was 12.4%, at the upper limit of the contemporary literature (4.5% to 12.4%) [3, 5, 7].

Notably, this stricture rate was significantly lower in patients in whom a visual obturator was used compared to the blind cohort. Furthermore, the blind obturator group exhibited an unusually high rate of acute urinary retention. This clinical finding likely reflects the higher incidence of unrecognized intraoperative mucosal edema, minor focal lacerations, or false passages resulting from mechanical friction during blind sheath advancement, which subsequently increased postoperative swelling and urinary retention. In addition, our study demonstrated that overall complication rates (including postoperative hematuria, urinary incontinence, erectile dysfunction, and urethral stricture) were lower in the visual obturator group.

Subgroup analyses based on prostate volume revealed that while urethral stricture was numerically less frequent in patients with prostate volumes ≤ 60 cc when a visual obturator was used, this difference did not reach statistical significance. In contrast, among patients with prostate volumes > 60 cc, the protective effect of the visual obturator became more pronounced and achieved statistical significance. This implies that the visual obturator technique is particularly essential for mitigating the prolonged mechanical stress associated with navigating larger prostates.

Regarding functional outcomes, both cohorts achieved statistically significant postoperative improvements in Qmax values. Interestingly, the absolute numerical increase in flow rate was greater in the blind cohort due to their lower baseline values. However, the visual obturator group demonstrated a more stable functional trend, showing significantly less reduction in Qmax between the 1-month and 6-month follow-up evaluations, indicating long-term functional stability.

Our study also evaluated the predictive capacity of traditional risk factors. While prior literature suggests prostate volume and operative time are important predictors of stricture formation, our ROC analysis showed that their individual clinical discriminatory power was modest (with AUC values close to 0.50), though operation time maintained a marginal significance in the blind group [2–6, 8]. Crucially, even this modest predictive value was completely mitigated in the visual obturator cohort, underscoring that utilizing an atraumatic introduction technique undermines the traditional risk impact of prolonged surgeries or larger prostate sizes.

Several limitations of this study should be acknowledged. First, despite utilizing propensity score matching, residual imbalances persisted between the two groups regarding BMI and smoking exposure. Although smoking and high BMI may theoretically impair microvascular perfusion and urethral tissue healing, our sub-analyses showed no direct statistical correlation between these variables and stricture formation within each group [3, 7].

Second, due to the multi-centric retrospective design, detailed data regarding stricture location, stricture length, and subsequent management strategies were not uniformly available across participating centers. Consequently, although urethral stricture incidence could be reliably assessed, the clinical severity and therapeutic implications of individual strictures could not be comprehensively evaluated. Future prospective studies incorporating standardized stricture characterization are warranted to better define the clinical impact of visual obturator use.

## Conclusion

In this large multicenter cohort, the use of a visual obturator for resectoscope sheath insertion during bipolar TURP was independently associated with a significantly lower incidence of postoperative urethral stricture and postoperative acute urinary retention. Advancement of the resectoscope under direct vision provided a clear protective effect, particularly in patients with larger prostate volumes (> 60 cc).

Notably, traditional predictors of urethral stricture lost their modest discriminatory value when a visual obturator was employed, underscoring the critical role of atraumatic instrument insertion. These findings suggest that routine use of a visual obturator represents a simple, low-cost, and highly effective strategy to minimize urethral trauma and improve procedural safety during TURP.

## Data Availability

All data generated or analyzed during this study are included in this article. Further enquiries can be directed to the corresponding author.

## Abbreviations

AUC: Area under the curve
BMI: Body mass ındex
BNC: Bladder neck contracture
BOO: Bladder outlet obstruction
BPH: Benign prostatic hyperplasia
CI: Confidence ınterval
LUTS: Lower urinary tract symptoms
PSA: Prostate-specific antigen
PSM: Propensity score matching
Qmax: Maximum urinary flow rate
ROC: Receiver operating characteristic
SD: Standard deviation
TURP: Transurethral resection of the prostate
US: Urethral stricture

## References

[1] Zeng XT, Jin YH, Liu TZ et al (2022) Clinical practice guideline for transurethral plasmakinetic resection of prostate for benign prostatic hyperplasia (2021 Edition). Mil Med Res 9(1):14. 10.1186/s40779-022-00371-6 35361280 10.1186/s40779-022-00371-6 PMC8974007

[2] Ashhad M, Khalil U, Abbasi S et al (2025) Therapeutic Efficacy and Complication Profile of Monopolar Transurethral Resection of the Prostate (TURP) in the Management of Bladder Outlet Obstruction. Cureus 17(8):e75432. 10.7759/cureus.75432 10.7759/cureus.89640 PMC12414532 40922865

[3] Wan Mokhter WM, Duan X, Yang J et al (2025) Construction of a nomogram to predict urethral stricture after transurethral resection of the prostate: A retrospective cohort study. PLoS ONE 20(2):e0313557. 10.1371/journal.pone.0313557 39937772 10.1371/journal.pone.0313557 PMC11819526

[4] Altıntaş E, Şahin A, Babayev H et al (2024) Machine learning algorithm predicts urethral stricture following transurethral prostate resection. World J Urol 42(1):335. 10.1007/s00345-024-05017-x 38748256 10.1007/s00345-024-05017-x PMC11096196

[5] Elsaqa M, Serag M, Leenlani N et al (2023) The incidence of urethral stricture and bladder neck contracture with transurethral resection vs. holmium laser enucleation of prostate: A matched, dual-center study. Can Urol Assoc J 17(1):E14–20. 10.5489/cuaj.8015 10.5489/cuaj.7967 PMC9872823 36121881

[6] Tao H, Jiang YY, Jun Q et al (2016) Analysis of risk factors leading to postoperative urethral stricture and bladder neck contracture following transurethral resection of prostate. Int Braz J Urol 42(2):302–311. 10.1590/S1677-5538.IBJU.2014.0500 27256185 10.1590/S1677-5538.IBJU.2014.0500 PMC4871391

[7] Afandiyev F, Ugurlu O (2022) Factors predicting the development of urethral stricture after bipolar transurethral resection of the prostate. Rev Assoc Med Bras (1992) 68(1):50–55. 10.1590/1806-9282.20210744 35239937 10.1590/1806-9282.20210550

[8] Porto JG, Bhatia AM, Bhat A et al (2024) Evaluating transurethral resection of the prostate over twenty years: a systematic review and meta-analysis of randomized clinical trials. World J Urol 42(1):634. 10.1007/s00345-024-05332-3 39547977 10.1007/s00345-024-05332-3 PMC11568034

[9] Kumar B, Srivastava A, Sinha T (2019) Urethral stricture after bipolar transurethral resection of prostate-truth vs hype: A randomized controlled trial. Indian J Urol 35(1):41–47. 10.4103/iju.IJU_114_18 30692723 10.4103/iju.IJU_223_18 PMC6334584

